# Identification and characterization of orthologs of *AtNHX5* and *AtNHX6* in *Brassica napus*

**DOI:** 10.3389/fpls.2012.00208

**Published:** 2012-09-11

**Authors:** Brett A. Ford, Joanne R. Ernest, Anthony R. Gendall

**Affiliations:** Department of Botany, La Trobe UniversityMelbourne, VIC, Australia

**Keywords:** Arabidopsis, NHX, antiporter, Brassica, sodium transport, potassium transport, pH, cation transport

## Abstract

Improving crop species by breeding for salt tolerance or introducing salt tolerant traits is one method of increasing crop yields in saline affected areas. Extensive studies of the model plant species *Arabidopsis thaliana* has led to the availability of substantial information regarding the function and importance of many genes involved in salt tolerance. However, the identification and characterization of *A. thaliana* orthologs in species such as *Brassica napus* (oilseed rape) can prove difficult due to the significant genomic changes that have occurred since their divergence approximately 20 million years ago (MYA). The recently released *Brassica rapa* genome provides an excellent resource for comparative studies of *A. thaliana* and the cultivated *Brassica* species, and facilitates the identification of *Brassica* species orthologs which may be of agronomic importance. Sodium hydrogen antiporter (NHX) proteins transport a sodium or potassium ion in exchange for a hydrogen ion in the other direction across a membrane. In *A. thaliana* there are eight members of the NHX family, designated *AtNHX1-8*, that can be sub-divided into three clades, based on their subcellular localization: plasma membrane (PM), intracellular class I (IC-I) and intracellular class II (IC-II). In plants, many NHX proteins are primary determinants of salt tolerance and act by transporting Na^+^ out of the cytosol where it would otherwise accumulate to toxic levels. Significant work has been done to determine the role of both PM and IC-I clade members in salt tolerance in a variety of plant species, but relatively little analysis has been described for the IC-II clade. Here we describe the identification of *B. napus* orthologs of *AtNHX5* and *AtNHX6*, using the *B. rapa* genome sequence, macro- and micro-synteny analysis, comparative expression and promoter motif analysis, and highlight the value of these multiple approaches for identifying true orthologs in closely related species with multiple paralogs.

## Introduction

It is estimated that more than 800 million hectares of land worldwide and approximately 20% of irrigated farmland are negatively impacted by salinity (FAO, 2009). While there are many different salts that contribute to the salinization of a landscape, by far the most abundant and damaging is NaCl (Tester and Davenport, 2003). NaCl in the soil inhibits plant growth by causing an initial osmotic stress, followed by the accumulation of Na^+^ ions in the plant to toxic levels (Munns and Tester, 2008). Improved crop yield under saline conditions can be achieved by identifying genes which confer salt tolerance, and introducing them to crop species by traditional breeding or transgenesis.

In plants, members of the monovalent cation/proton antiporter (CPA1) gene family exchange a sodium, potassium or lithium ion for a hydrogen ion across a cellular membrane. They can be classified into two distinct sub families, the plasma membrane (PM) localized NHAP family and the intracellular localized (IC) NHX family (Brett et al., 2005a; Chanroj et al., 2012). These proteins are important in maintaining pH and ion homeostasis, and are conserved across all phyla and kingdoms (Brett et al., 2005a). In *Arabidopsis thaliana*, there are two members of the NHAP family (SOS1/AtNHX7 and AtNHX8) and six members of the IC-NHX family, designated AtNHX1-6 (Brett et al., 2005a; Chanroj et al., 2012). The IC-NHX family can be further sub-divided into two clades based on their cellular localization: the IC-I clade (*AtNHX1-4*), localized to the tonoplast; or the IC-II clade (*AtNHX5-6*), localized to endosomal compartments (Brett et al., 2005a; Bassil et al., 2011). In plants, NHX proteins are important determinants of Na^+^ tolerance, detoxifying cytosolic Na^+^ by transportation out of the cell to the apoplastic space, or by sequestration into subcellular compartments (Apse et al., 1999; Shi et al., 2003; Rodriguez-Rosales et al., 2008). Members of the PM (*AtNHX7/SOS1*) and IC-I (*AtNHX1*) clades have been shown to be upregulated in response to NaCl, and to confer salt tolerance upon over-expression in *A. thaliana* (Apse et al., 1999; Shi et al., 2002, 2003; Shi and Zhu, 2002). Of the IC-II clade, *AtNHX5* and the rice ortholog *OsNHX5* are both up regulated in response to NaCl (Yokoi et al., 2002; Fukuda et al., 2011) and over expressing *AtNHX5* in a variety of plant species including rice (Li et al., 2011) and tomato (Rodriguez-Rosales et al., 2008) has been shown to increase their tolerance to NaCl. Recent work has shown that *AtNHX5* and *AtNHX6* are functionally redundant, are important for normal plant growth and development, are endosome associated and have an important role in protein trafficking to the vacuole (Bassil et al., 2011). A similar role in protein trafficking has been demonstrated for the yeast ortholog ScNHX1 (Bowers et al., 2000; Brett et al., 2005b). Interestingly, comparatively little work has been undertaken investigating the role of the Class II NHX proteins from important crop species in NaCl tolerance.

**Figure 1:**
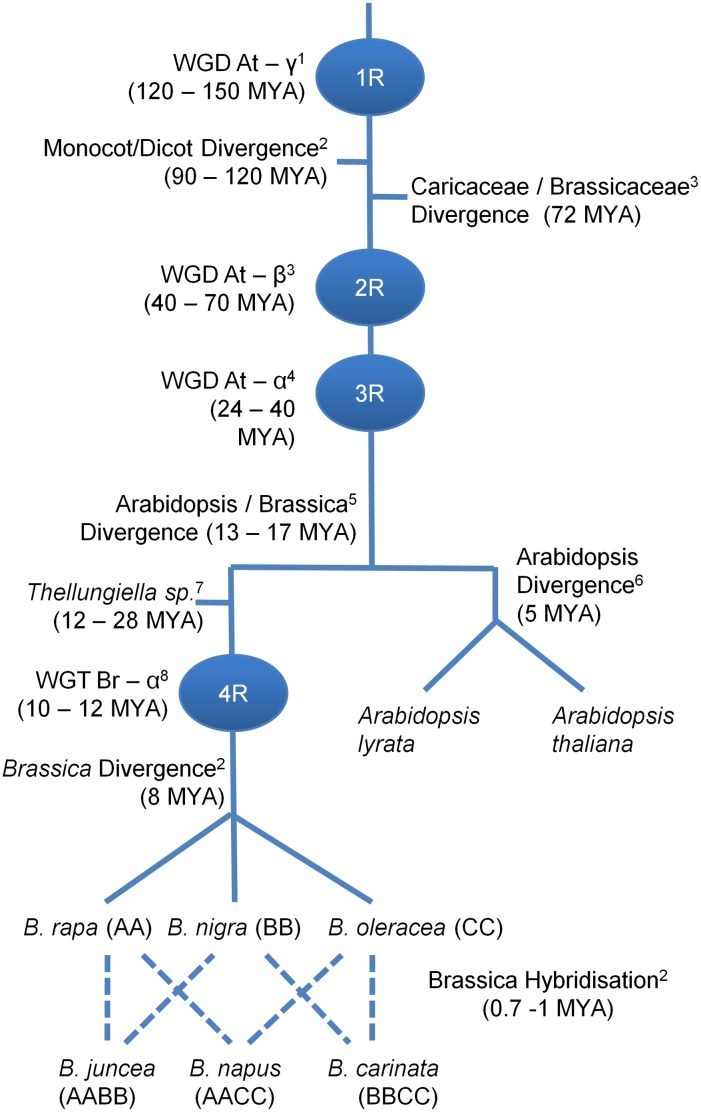
**Genome evolution of *B. napus* and *A. thaliana.*** Prior to their divergence approximately 13–17 MYA *B. napus* and *A. thaliana* share a complex genome evolution including three (denoted 1R, 2R, and 3R) whole genome duplication (WGD) events. The last two WGD events post date the divergence of the Moncots/Dicots and the Caricaceae/Brassicaceae. Since their divergence the ancestor of extant Brassica species underwent a whole genome triplication event (WGT, denoted 4R) with the allopolyploid *B. napus* arising from the hybridization of *B. rapa* and *B. oleracea*. Superscript numbers relate to references for divergence times. For an in depth analysis of evolutionary history of Brassicaceae please refer to the excellent review by Franzke et al. (2011). ^1^(Soltis et al., 2009); ^2^(Mun et al., 2009); ^3^(Bell et al., 2010); ^4^(Henry et al., 2006); ^5^(Yang et al., 1999); ^6^(Koch et al., 2000); ^7^(Couvreur et al., 2010); ^8^(Lysak et al., 2005).

*Brassica napus* is a member of the Brassicaceae family and is an important oil crop species. *B. napus* cultivation has increased significantly over recent years and it is now the second largest crop for the production of oil seeds and oil meals, and the third largest crop used in the production of vegetable oil (USDA, 2009). As both *A. thaliana* and *B. napus* are members of the family Brassicaceae and therefore relatively closely related, a comparative study could be undertaken to try to identify the homologs of *AtNHX5* and *AtNHX6* to assess their importance in salt tolerance. However, there have been extensive large- and small-scale genome changes in the *A. thaliana* and *B. napus* genomes since their divergence approximately 13–17 million years ago (MYA) (Figure 1). *B. napus* is an allopolyploid species (AACC, 2*n* = 38) arising from the hybridization of *B. rapa* (AA, 2*n* = 20) and *Brassica oleracea* (CC, 2*n* = 18) (Figure 1). Additionally, all *Brassica* species appear to have undergone a whole genome triplication (WGT, Br—α) event since they diverged from *A. thaliana* (Figure 1). The *A. thaliana* genome has undergone extensive duplications, deletions, re-arrangements and a reduction in chromosome number even since its divergence from its close relative *Arabidopsis lyrata* only 5 MYA (Figure 1) (Hu et al., 2011). These confounding factors make direct comparative studies and homolog identification in *B. napus* based solely on *A. thaliana* genomic information problematic.

To overcome this problem, Schranz et al. (2006), building on the work of Parkin et al. (2005), have proposed that the comparative studies of *A. thaliana* and the *Brassica* species should be based upon an ancient karyotype of *n* = 8, that is observed in at least 37% of the Brassicaceae species. The genome of this ancient karyotype can be further segmented into 24 conserved syntenic blocks (designated A–X) that can be identified in both the *A. thaliana* genome and the *B. rapa* (A genome) component of *B. napus* (Parkin et al., 2005; Schranz et al., 2006).

While this approach greatly aided comparative studies between *A. thaliana* and *B. napus*, the lack of high quality *Brassica* sequence still inhibited homolog identification. The recent release of the fully sequenced *B. rapa* genome (Wang et al., 2011) has enabled in-depth comparative studies between *A. thaliana* and *B. napus*.

Analysis of the *B. rapa* genome reveals that there has been significant gene loss since the most recent WGT (Wang et al., 2011). The triplicated *B. rapa* genome can be divided into three sub genomes based on their level of gene loss relative to *A. thaliana*, which have been designated the least fractionated genome (LF) with 30% gene loss, the medium fractionated genome (MF1) with 54% gene loss and the most fractionated genome (MF2) with 64% gene loss (Wang et al., 2011). However, analysis of the *B. rapa* genome shows that genes associated with response to environmental factors such as salt were over-retained (Wang et al., 2011) and as a result it is possible that there are three homologs of *AtNHX5* and three homologs of *AtNHX6* in *B. rapa.* As *B. napus* has both a *B. rapa* triplicated genome, and a triplicated *B. oleracea* C genome component there may be up to six *AtNHX5* and six *AtNHX6* homologs.

Here we describe the use of the *B. rapa* genomic sequence to aid in the clear identification of the *B. napus* homologs of *AtNHX5* and *AtNHX6* as a first step in evaluating their potential to improve salt tolerance.

## Materials and methods

### Identification and reannotation of *B. rapa NHX* genes

The initial identification of the complete set of *NHX* genes in *B. rapa* used representative *A. thaliana* NHX protein sequences (See Table 1 for accession numbers) as query sequences in TBLASTN searches of the *B. rapa* genomic database in Phytozome version 8.0 (http://www.phytozome.org; Goodstein et al., 2012). Initial searches were performed using AtNHX1, AtNHX6, and AtSOS1/AtNHX7 amino acid sequences. Hits with an *E*-value of less than 10^−45^ were classified as putative NHXs and examined further. Preliminary alignments of *A. thaliana* NHX proteins to the annotated *B. rapa* proteins revealed some minor sequence dissimilarities in annotations that often corresponded to the lack of one or more exons in the *B. rapa* gene models, or from fusion of two adjacent genes into a single mis-annotated *BrNHX* gene. To refine these predictions, two approaches were used. Expressed sequence tag (EST) or cDNA sequences were identified by using the NHX amino acid sequences as the query for a TBLASTN search of all EST databases restricting the search to the taxid *Brassica*. The EST or cDNA sequences were then assigned to the relevant *NHX* gene based on the regions of maximum match and sequence alignment to the corresponding *B. rapa* genome. These sequences were aligned with the corresponding genomic region using Spidey (http://www.ncbi.nlm.nih.gov/IEB/Research/Ostell/Spidey/index.html), using the “Plant” genomic sequence option and other default options. The identified exons were then incorporated into a final re-annotated open reading frame model. If no EST or cDNA support existed, amino acid sequences from the *A. thaliana* or other *B. rapa* ortholog corresponding to the mis-annotated regions were used as queries in a low stringency TBLASTN approach (expected *E*-value of 1.0), which allowed the correction of the original annotations. Gene models for *BrNHX6.1* and *BrNHX6.2* were generated using the FancyGene 1.4 gene model drawing program (http://www.bio.ieo.eu/fancygene Rambaldi and Ciccarelli, 2009). *B. rapa NHX* gene locations were determine by using the Brassica Database (http://brassicadb.org/brad).

**Table 1:** **Chromosomal locations of *B. rapa NHX* genes**.

***A. thaliana gene name***	**AGI**	***A. thaliana genomic segment^a^***	**Corresponding *B. rapa segment locations^b^***	**Br gene name**	***B. rapa (scaffold, fractionated genome^c^)***	**Location (ATG-STOP)**	***Brassica sp. ESTs***
NHX1	AT5G27150	Q	2, 6, 9	BrNHX1.1	Bra020599 (A02, MF2)	24295626–24298768	*B. juncea* HQ848296, HQ848294, HQ848295
–	–	–	–	BrNHX1.2	Bra036110 (A09, MF1)	2662958–2665986	*B. rapa* EX098258.1; *B. napus*: GU192449.1
–	–	–	–	BrNHX1.3	Bra009975 (A06, LF)	~18416170–~18415490	None (and likely pseudogene)
NHX2	AT3G05030	F	1, 3, 5	BrNHX2	Bra039469 (A05, LF)	23480049–23483092	*B. napus* AY189676.1
NHX3	AT5G55470	C	5, 6, 8	BrNHX3	Bra002905 (A10, LF)	6853381–6850500	None
NHX4	AT3G06370	F	1, 3, 5	BrNHX4	Bra020755 (A02, LF)	23272879–23269821	None
NHX5	AT1G54370	C	5, 6, 8	–	Not detected	–	None
NHX6	AT1G79610	E	2, 7, 7	BrNHX6.1	Bra035130 (A07, LF)	22302738–22298998	*B. oleracea* DK554651.1; DK541204.1; DK548975.1; DK489391.1
–	–	–	–	BrNHX6.2	Bra003601 (A07, MF2)	14001071–14007892	*B. napus* FG553917.1; ES983056.1: CD827425.1; *B. oleracea* EE531575.1; EE530829.1; DK495073.1; DK481606.1
NHX7/SOS1	AT2G01980	K	2, 6, 9	BrSOS1	Bra017430 (A09, MF2)	15259302–15253531	*B. rapa* HQ848290; *B. napus* EU487184.1; *B. juncea* HQ848289, HQ949287, HQ848288, EF206779
NHX8	AT1G14660	A	6, 8, 9, 10	BrNHX8	Bra026197 (A06, LF)	5641695–5637360	None

a A. thaliana chromosomal blocks as described in Schranz et al. (2006).

b Numbers of B. rapa segments were as described in Wang et al. (2011).

c B. rapa chromosomal location and sub-genome were assigned using the Brassica Database (http://brassicadb.org/brad).

### Identification of *Thellungiella parvula NHX* genes

The identification of the intra-cellular *NHX* genes in *T. parvula* used representative Arabidopsis NHX protein sequences (See Table 1 for accession numbers) as query sequences in BLASTP searches of the *T. parvula* predicted gene models—protein version 2.0 database in the *Thellungiella* website [http://www.phytozome.org; (Dassanayake et al., 2011; Goodstein et al., 2012)]. Initial searches were performed using AtNHX1 and AtNHX6 amino acid sequences. Hits with an E value of less than 10^−45^ were classified as putative NHXs and examined further by phylogentic analysis.

### Phylogenetic analysis

The predicted amino acid sequences of all predicted *B. rapa, T. parvula* and *A. thaliana* NHXs were imported into MEGA5 (Tamura et al., 2011), and aligned using ClustalW with default parameters, and the corresponding amino acid alignments used for subsequent analyses. A neighbor-joining analysis was performed, using the Poisson model of amino acid substitution, with uniform rate among sites and pairwise deletion, and the phylogeny tested with 1000 bootstrap replications.

NHX6 nucleotide sequences from *B. rapa*, *B. oleracea* and *B. napus* and *Brassica* NHX6 ESTs from Genbank were imported into MEGA5 (Tamura et al., 2011) and aligned using Clustal W with default parameters. A neighbor-joining analysis was performed using the maximum composite likelihood method with uniform rate among sites and complete deletion, and the phylogeny tested with 1000 bootstrap replications.

### Microsynteny of *AtNHX5* and *AtNHX6* and their co-linearity in *B. rapa*

*AtNHX5* and *AtNHX6* sequences were used as the query to search for homeologous regions in the three sub genomes in *B. rapa* using the syntenic paralogs search tool in the Brassica Database (http://brassicadb.org/brad). The region of *A. thaliana* chromosome 1 containing *AtNHX6* and eight flanking genes upstream and downstream was then aligned to the three co-linear genomic segments from *B. rapa*. The region of *A. thaliana* chromosome 1 surrounding *AtNHX5* was aligned in a similar manner, however due to the more complex nature of the co-linearity between this region and the corresponding *B. rapa* genomic segments the region of analysis was expanded to include 14 genes downstream and 12 genes upstream of *AtNHX5*.

### *BrNHX6* promoter analysis

Nucleotide sequences of *AtNHX6*, *BrNHX6.1*, and *BrNHX6.2*, including 1119 bp upstream of the predicted ATG start codon, and 500 bp downstream of the predicted stop codon were obtained from Phytozome. Sequences were aligned and subjected to sliding window analysis of homology using VISualization Tool for Alignments (VISTA) web-based software (Frazer et al., 2004). Regions identified as highly conserved were analysed for conserved *cis*-regulatory elements (CRE) motifs using the Plant cis-acting regulatory DNA elements (PLACE) database (Higo et al., 1999).

### Plant growth conditions

Seeds of *B. rapa* (cv Chinese cabbage), *B. oleracea* (cv White cabbage) and *B. napus* (cv Westar) were sown direct to soil in seed raising mix and vermiculite (3:1) and watered twice a week. A general purpose fertilizer (Osmocote) was applied in liquid form every two weeks and plants were grown in a 16 h light: 8 h dark photoperiod at 22°C.

For material used for qRT-PCR and isolation of cDNA, *B. napus* (cv Westar) seeds were surface sterilized by soaking in 70% ethanol for 5 min and 1% commercial bleach for 10 min. Seeds were washed three times in sterile double distilled water and plated onto half strength MS plates (Murashige and Skoog, 1962) with 0 mM or 200 mM NaCl. Three replicate plates were made for each treatment. Seeds were stratified for 72 h and grown in a 16 h light: 8 h dark photoperiod at 22°C.

### Cloning of brassica NHX6 orf sequences

Genomic DNA was extracted from *B. rapa* (cv Chinese cabbage) as described (Herrmann and Frischauf, 1987). RNA was extracted from eight week old leaf material of *B. napus* (cv Westar) and *B. oleracea* (cv white cabbage) and from two week old whole seedlings *B. napus* (cv Westar) using the Qiagen RNeasy Kit (Qiagen #74104) as per manufacturer's instructions. Approximately 500 ng of RNA was used to synthesize cDNA using Superscript III (Life Technologies #18080) following the manufacturer's recommendations, and the cDNA was diluted to a final volume of 100 μL with sterile double distilled water.

Full length *Brassica* NHX6 ORF sequences were amplified by PCR from cDNA derived from eight week old leaf material of *B. oleracea* (Forward 5′-CACCATGTCGGAGATTTCGCCG-3′ and Reverse 5′-TAACCGGGGGCTAAATTTCTGA-3′) and *B. napus* (Foward 5′-CACCATGTCGGAGATTTCGCCG-3′ and Reverse 5′-TAACCGGGGGCTAAATTTCTGA-3′). The PCR product was then cloned into the pENTR D/TOPO vector (Life Technologies #K2435-20) as per manufacturer's instructions. Approximately 10 independent clones from each ligation were sequenced with one unique coding sequence being identified for both *B. napus* (Genbank JX082291) and *B. oleracea* (Genbank JX082292).

### Identification and cloning of partial *Brassica* NHX6 sequences

#### Primer design

The *B. rapa NHX6.1* and *NHX6.2* genes, the full length *B. napus* (JX082291) and *B. oleracea* (JX082292) ORF sequences as well as other *B. napus*. *B. rapa* and *B. oleracea* EST sequences that were available were aligned in Vector NTI v11.5 (Invitrogen). From this alignment regions of 100% nucleotide conservation between all divergent *Brassica* species sequences were identified and primers (Forward 5′-GCTTGAAGCCCTAGAGGTTGT-3′ and Reverse 5′-CGTTATTACTTGTGAAGAACGTGTT-3′) designed within these regions to amplify all potential A and C genome *B. napus* NHX6 homologs.

Using these primers, partial *Brassica* NHX6 sequences were amplified by PCR from cDNA of 2-week-old whole seedlings in *B. napus* and from genomic DNA in *B. rapa*. DNA from the PCR was purified using the Promega Wizard PCR clean up kit (Promega #A9282) and then cloned into pGEM T-easy vector (Promega #A1360) as per manufacturer's instructions. Approximately 10 independent clones from each ligation were sequenced using the M13_F sequencing primer.

### Gene expression analysis

Total RNA was extracted from whole seedlings of *B. napus* (cv Westar) two weeks after germination using the Qiagen RNeasy Kit (Qiagen #74,104) as per manufacturer's instructions. Total RNA (2 μg) was DNase treated with the Promega RQ1 DNase kit (Promega #M6101) as per manufacturer's instructions to remove any genomic DNA contamination.

Primers were designed for the *BnNHX6.1* gene (Forward 5′-CATCCTTTTCTCATTCTGTTCATCG-3′ and Reverse 5′-TCGAAGTCCACTGTACCAAAG-3′) and for the *BnNHX6.2* gene (Forward 5′-GATAGCCGTGATACATCCCTTG-3′ and Reverse 5′-AGTTCTGAAAATGACTTTGCGC-3′) based on the corresponding BrNHX6.1 or BrNHX6.2 open reading frame sequences identified from the *B. rapa* genome.

Quantitative real-time PCR was performed using the Bio-Rad iCycler and the iScript One Step RT-PCR kit with SYBR green (Bio-Rad #170-8892) as per manufacturer's instructions. PCR conditions consisted of a reverse transcription step at 50°C for 10 min, a reverse transcription inactivation step at 95°C for 5 min and 40 cycles of 95°C for 10 s followed by either 61°C (*BnNHX6.1*) or 60°C (*BnNHX6.2*) for 30 s. Results were visualized using BioRad iQ5 Optical System Software. A dissociation analysis was performed and the PCR products were sequenced to confirm PCR specificity to the *BnNHX6* transcript. Expression of the *BnNHX6* genes was normalized relative to the expression of BnUBC_21 (Forward 5′-CCTCTGCAGCCTCCTCAAGT-3′ and Reverse 5′-CATATCTCCCCTGTCTTGAAATGC-3′) previously validated as a reference gene for qRT-PCR in *B. napus* (Chen et al., 2010). Transcript abundance was calculated using the Pfaffl model for relative quantification with efficiency correction (Pfaffl, 2004). All experiments were completed in triplicate on three independent biological replicates.

## Results

### Identification and phylogenetic analysis of *B. rapa NHX* genes

**Figure 2:**
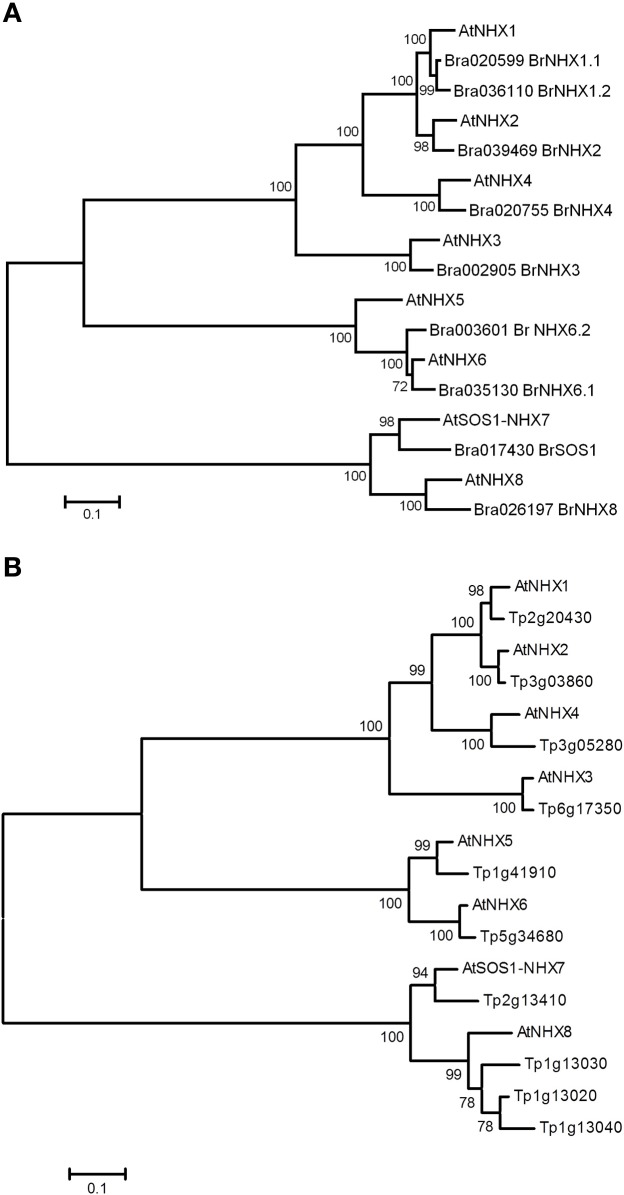
**Phylogenetic analysis of *B. rapa* and *T. parvula* NHX proteins.** Amino acid sequences of NHX proteins from *B. rapa* **(A)**, *T. parvula* **(B)** and *A. thaliana* were aligned using Clustal W, and a Neighbor-joining tree generated. Numbers at nodes correspond to bootstrap support percentages following 1000 replications, and the scale is in amino acid changes per site.

As a first step to identify the *B. napus AtNHX5* and *AtNHX6* homologs, we searched the now fully sequenced *B. rapa* genome. As *AtNHX5* and *AtNHX6* are very closely related to the other *A. thaliana* NHX members *AtNHX1-4* and *AtNHX7-8*, unmabiguous identification of the corresponding *B. rapa* homologs may be difficult. To clearly identify and unambiguously assign the *AtNHX5* and *AtNHX6 B. rapa* homologs, we identified all the *NHX* genes in the genome of *B. rapa*. To determine the complete complement of *NHX* genes, we searched the *B. rapa* genome sequence and identified ten *NHX* genes (hereafter called *BrNHX;* see Table 1 for complete details). A phylogenetic analysis of the corresponding predicted amino acid sequences of the encoded proteins revealed that *B. rapa* is likely to express five intracellular class one (IC-I) genes, two intracellular class two (IC-II) genes and two PM clade members (Figure 2A). Additional evidence of one pseudogene (*BrNHX1.3*) could also be detected. In some cases, multiple *B. rapa* genes could be detected that corresponded to a single *NHX* gene in *A. thaliana*, but surprisingly, in only one case could three *B. rapa* orthologs corresponding to a *AtNHX* gene be detected (*AtNHX1* and *Br.NHX1.1, Br NHX1.2* and *BrNHX1.3*; Table 1) suggesting substantial gene loss of *NHX* genes in *B. rapa* following the most recent WGT. To further investigate this gene loss, we examined the chromosomal location of the *B. rapa NHX* genes, and compared these to the described fractionated genomes (Wang et al., 2011). Six of the 10 *B. rapa* NHXs were retained on the LF sub-genome, one on the MF1 and three on the MF2 (Table 1). The proteins encoded by the two class II-IC *NHX* genes identified in *B. rapa* are clearly differentiated from other members of the *A. thaliana* NHX family (Figure 2A) and are likely to be the true orthologs of *AtNHX5* and *AtNHX6*. Interestingly, both of the identified class II-IC *NHX* genes are more closely related to *AtNHX6* than to *AtNHX5* (Figure 2A) indicating that there may not be a *B. rapa* ortholog of *AtNHX5*.

To further investigate the apparent absence of a *AtNHX5* ortholog in *B. rapa*, we decided to try to identify the intra-cellular *NHX* genes in the closely related species *T. parvula. T. parvula* diverged from the *Brassica* species after the divergence from *A. thaliana* but before the Br—α WGT event (Figure 1). Six intra-cellular *NHX* genes were identified and the phylogentic analysis showed that there was one unique ortholog of each of the *A. thaliana NHX1-6* genes (Figure 2B), indicating that there was an *AtNHX5* ortholog present in the ancient *Brassica* ancestor species before the Br—α WGT.

### Macro- and micro-synteny of *AtNHX5 and AtNHX6*

**Figure 3:**
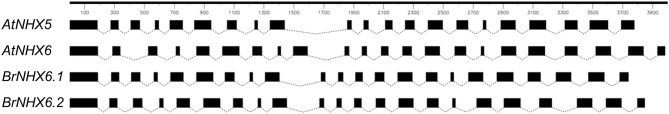
**Predicted gene models for AtNHX5, AtNHX6, BrNHX6.1 and BrNHX6.2.** Predicted gene models for AtNHX5, AtNHX6, BrNHX6.1, and BrNHX6.2. Exons are shown as black boxes and introns are shown as dotted lines. Gene models were generated using FancyGene 1.4 (http://www.bio.ieo.eu/fancygene).

To be confident that the *BrNHX6.1* and *BrNHX6.2* genes identified are both orthologs of *AtNHX6* and not *AtNHX5*, we analysed the macrosynentic relationship between these genes based on the 24 conserved syntenic blocks between *A. thaliana* and *B. rapa* (Schranz et al., 2006). Analysis of the conserved co-linearity in *A. thaliana* and *B. rapa* shows that *BrNHX6.1*, *BrNHX6.2*, and *AtNHX6* are all located in the retained conserved genomic block E and are likely to be true orthologs, whereas *AtNHX5* is retained within the conserved genomic Block C on the *A. thaliana* genome (Table 1), and appears to have no clear ortholog in *B. rapa*. We also analysed the gene models for *AtNHX5, AtNHX6, BrNHX6.1*, and *BrNHX6.2* (Figure 3). This showed that *AtNHX6, BrNHX6.1*, and *BrNHX6.2* all have a common 22 exon/21 intron gene structure, whereas *AtNHX5* has only 21 exons (Figure 3) supporting the notion that the *BrNHX6.1* and *BrNHX6.2* genes are both orthologous to *AtNHX6* and not *AtNHX5.*

**Figure 4:**
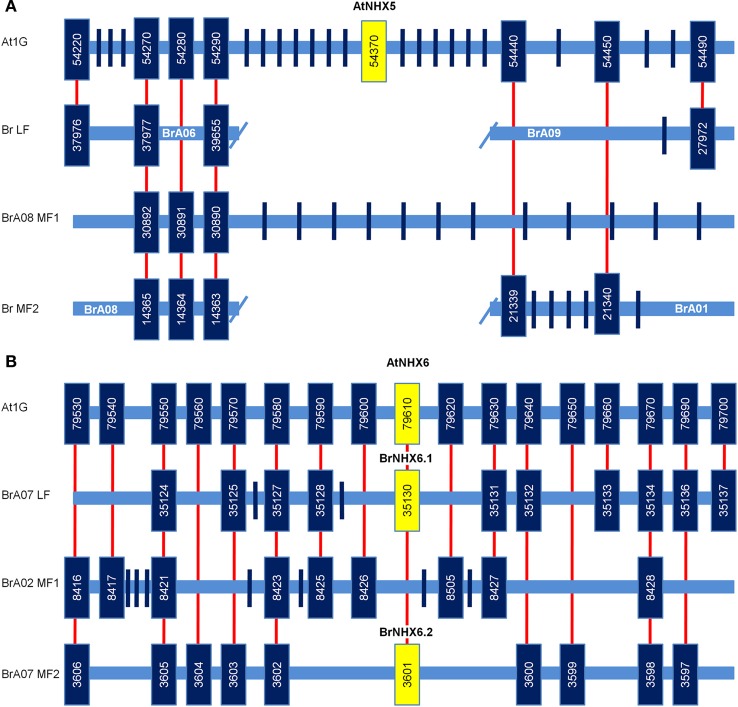
**Microsynteny of AtNHX5 and AtNHX6 and conserved co-linearity in *B. rapa*.** Comparison of co-linearity between AtNHX5 **(A)** and AtNHX6 **(B)** (highlighted in yellow) in the Arabidopsis genome and the least fractionated (LF), medium fractionated (MF1) and most fractionated (MF2) sub-genomes in *B. rapa*. The boxes and bars denote annotated genes (boxes contain gene accession number) and the red lines connect syntenic homologous genes (Note corresponding genomic regions are not drawn to scale).

Although it is clear that there was an AtNHX5 ortholog present prior to the Br—α WGT event, no *AtNHX5* homolog was identified in the *B. rapa* genome. To determine the cause of this discrepancy we decided to carefully investigate the microsynteny around *AtNHX5* (Figure 4A). The microsynteny in the *A. thaliana* genome, including fourteen genes downstream and twelve genes upstream of *AtNHX5*, was compared to the corresponding co-linear sub genome regions in the *B. rapa* genome (Figure 4A). Surprisingly, the corresponding co-linear regions of the three *B. rapa* sub genomes contain only five (LF), three (MF1) and nine (MF2) genes respectively, and only four (LF), three (MF1) and five (MF2) of these genes were homologous to *A. thaliana* genes (Figure 4A). The colinearity between *A. thaliana* and the *B. rapa* sub genomes is relatively well conserved from the At1G54220 gene to At1G54290 (Figure 4A). However, the next 14 genes upstream of At1G54290 in the *A. thaliana* genome including *AtNHX5* are completely absent from all three co-linear *B. rapa* sub genomes (Figure 4A). Co-linear genes are again found between the *A. thaliana* genome and the *B. rapa* LF sub genome from At1G54490 and the MF2 sub genomes from At1G5440, however, no homologous genes can be detected in the MF1 sub genome (Figure 4A). Interestingly, upstream of At1G54440 the co-linear region on the LF sub genome is found on chromosome BrA09, whereas the co-linear region of the LF sub genome downstream of At1G54290 was found on chromosome BrA06 (Figure 4A). Similarly, the region of co-linearity upstream of At1G54440 on the MF2 sub genome is found on chromosome BrA01 and the co-linear region of the MF2 sub genome downstream of At1G54290 was found on chromosome BrA08 (Figure 4A). This indicates that the syntenic regions around *AtNHX5* in the LF and MF2 sub genomes have probably undergone an inter-chromosomal translocation event. It also appears that there has been a large fragment deletion event in the MF1 sub genome in the syntenic region around *AtNHX5*. These chromosomal re-arrangments and deletions would account for the absence of any *AtNHX5* orthologs in *B. rapa* and may be the result of the genome reduction processes post the Br—α WGT event. This evidence, combined with the inability to identify any *AtNHX5* orthologs when searching the *B. rapa* genome, strongly suggest that there are no *AtNHX5* orthologs present in *B. rapa*.

The microsynteny in the *A. thaliana* genome, including eight genes downstream and eight genes upstream of *AtNHX6*, was compared to the corresponding co-linear sub genome regions in the *B. rapa* genome (Figure 4B). The corresponding three co-linear *B. rapa* sub genomes contain 13 (LF), 16 (MF1), and 10 (MF2) genes, respectively, with 11 (LF), nine (MF1), and 10 (MF2) homologous genes to the co-linear region in the *A. thaliana* genome (Figure 4B). The *BrNHX6.1* gene is located on the LF sub genome and the *BrNHX6.2* gene is located of the MF2 sub genome, but there is no *AtNHX6* ortholog present on the MF1 sub genome (Figure 4B). Importantly, all of the 17 *A. thaliana* genes in the region surrounding *AtNHX6* have at least one homolog in one of the corresponding *B. rapa* sub genomes and the order of the genes in *A. thaliana* is maintained within *B. rapa* (Figure 4B). The high degree of co-linearity between the region in *A. thaliana* genome containing *AtNHX6* and the corresponding regions in the *B. rapa* genome strongly support the notion that there are only two *AtNHX6* orthologs in *B. rapa* with the third ortholog likely to have been lost from the MF1 sub genome since the last whole genome triplication event.

### Promoter analysis of BrNHX6.1, BrNHX6.2 and AtNHX6

Although the *B. rapa* genome does not appear to contain a homolog to *AtNHX5*, there is a high level of genetic conservation between the *A. thaliana* and *B. rapa NHX6.1* and *NHX6.2* orthologs. To explore the potential for differential functionality between these two genes, we investigated the level of sequence similarity between their respective promoter regions and the conservation of specific *cis*-acting regulatory motifs.

**Figure 5:**
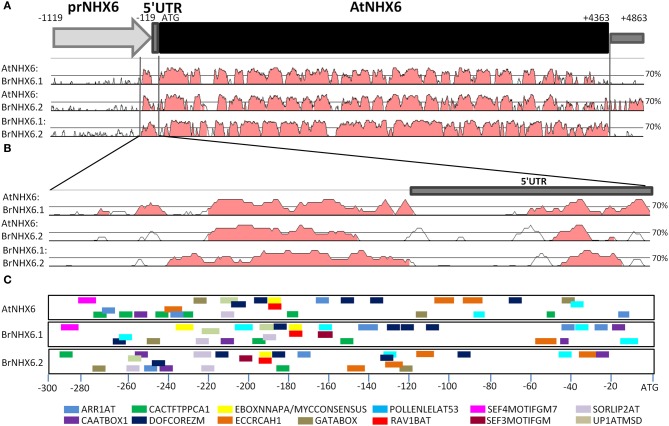
**Conservation of promoter regions of NHX6 homologues in *B. rapa*.** Graphic output for comparisons of *Arabidopsis* and *B. rapa* IC II NHX genome region **(A)** and putative promoter region **(B)** using VISTA, with parameters of at least 70% identity within a window of 25 b. Regions with >70% identity shared by the three orthologs are shaded in pink. **(C)** Positions of known plant *cis*-acting regulatory elements which are common to all three genes are shown as colored bars.

To identify conserved regions within the promoters of *B. rapa NHX6* orthologs, we compared nucleotide sequences of *AtNHX6*, *BrNHX6.1*, and *BrNHX6.2* (Figure 5A). The gene sequences, including 1119 bp upstream and 500 bp downstream, were subjected to sliding-widow analysis of homology. It has previously been shown that requiring 70% identity within a 25-bp window returns the greatest number of regulatory elements with acceptable specificity (Guo and Moose, 2003). Using these parameters, sliding window analysis revealed a highly conserved region of approximately 300 bp immediately upstream of the start codon, including the 5′UTR (Figure 5B).

Evaluation of this highly conserved promoter region (−300 bp) using the PLACE database SIGNALSCAN tool identified 10 *cis*-acting regulatory elements (CREs) which were common to all three genes: ARR1AT (5′-NGATT-3′), CAATBOX1 (5′-CAAT-3′), CACTFTPPCA1 (5′-YACT-3′), DOFCOREZM (5′-AAAG-3′), EBOXNNAPA (5′-CANNAG-3′), EECCRCAH1 (5′-GANTTNC-3′), GATABOX (5′-GATA-3′), MYCCONSENSUS (5′-CANNAG-3′), POLLENLELAT53 (5′-AGAAA-3′), RAV1BAT (5′-CACCTG-3′), SORLIP2AT (5′-GCCAC-3′), and UP1ATMSD (5′-GGCCCAWWW-3′). Additio-nally, the functionally similar elements SEF4MOTIFGM7S (5′-RTTTTTR-3′) and SEF3MOTIFGM (5′-AACCCA-3′) were conserved between *AtNHX6*:*BrNHX6.1* and *BrNHX6.1*:*BrNHX6.2*, respectively. The position of these motifs in the promoter sequence relative to the start codon is depicted in Figure 5C. Interestingly, the position of the DOFCOREZM (5′-AAAG-3′) and MYCCONSENSUS/EBOXNNAPA (5′-CANNAG-3′)/RAV1BAT (5′-CACCAG-3′) elements are conserved across the three promoter regions.

### Identification *B. napus* NHX6 homologs

**Figure 6:**
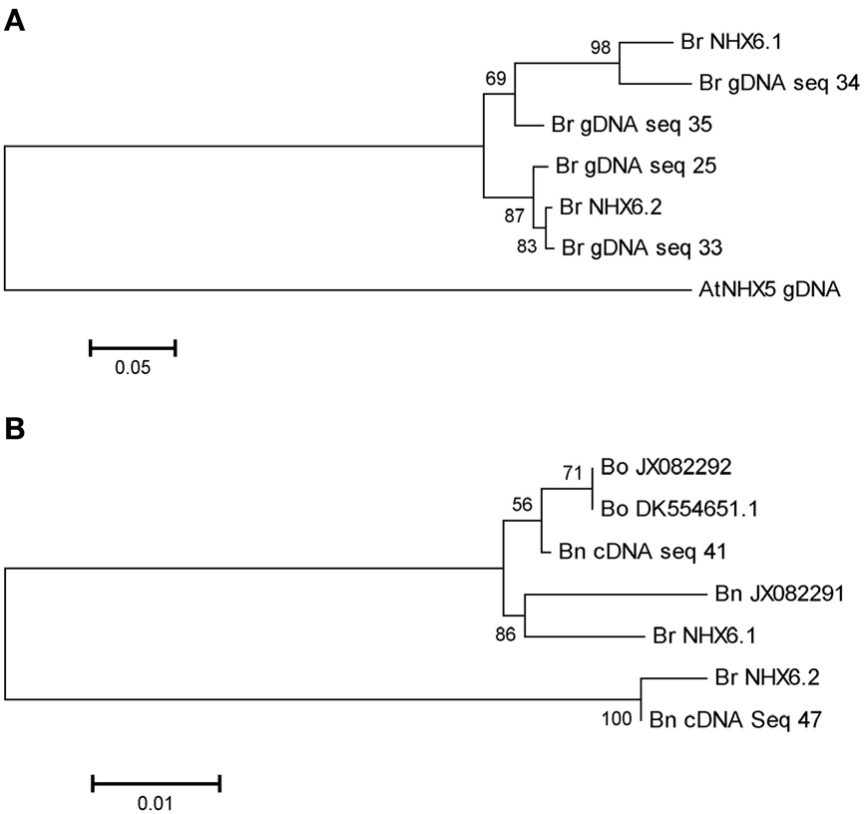
**Phylogenetic analysis of *B. rapa*, *B. oleracea*, and *B. napus* NHX6 partial sequences.** *B. rapa* gDNA **(A)** and *B. rapa*, *B. oleracea* and *B. napus* mRNA **(B)** sequences were aligned using Clustal W, and a Neighbor-joining tree generated. Numbers at nodes correspond to bootstrap support percentages following 1000 replications.

*B. napus* is an agriculturally important member of the Brassicaceae family. Due to the significance of salinity in *B. napus* cultivation, we sought to identify the complement of expressed *B. napus NHX6* homologs and assess their role in salt tolerance. To identify *NHX6* homologs in *B. napus* without the advantage of a complete *B. napus* genome sequence, we designed primers in absolutely conserved regions present in all available nucleotide sequences from *B. napus*, *B. rapa*, and *B. oleracea*. These primers were first tested on *B. rapa* genomic DNA to ensure they could identify both *BrNHX6.1* and *BrNHX6.2*. Using these “universal” *Brassica* NHX6 primers, four unique sequences were identified in *B. rapa.* A phylogenetic analysis of these sequences revealed two distinct groups including either the *BrNHX6.1* or the *BrNHX6.2* sequences from the *B. rapa* genome (Figure 6A). The presence of two unique sequences grouping with each *BrNHX6* sequence is most likely the result of heterozygous *BrNHX6* alleles present in the *B. rapa* genomic DNA tested. Importantly, however, the primers used were able to amplify both the *BrNHX6* homologs.

The complement of expressed *AtNHX6* homologs in *B. napus* was then investigated using the same approach, but using mRNA isolated from *B. napus* seedlings. Sequencing the cloned amplicons from *B. napus* cDNA identified only two unique sequences. A phylogenetic analysis was performed including *BrNHX6.1* and *BrNHX6.2* sequences from the *B. rapa* genome, the *B. napus* (JX082291) and *B. oleracea* (JX082292) ORF sequences and a *B. oleracea* (DK554651) EST sequence. The phylogenetic analysis showed two major groupings, each containing either the *BrNHX6.1* or the *BrNHX6.2* sequence (Figure 6B). Interestingly, only one sequence grouped with *BrNHX6.2* while four unique sequences grouped with *BrNHX6.1* (Figure 6B). The sequences that grouped with *BrNHX6.1* were divided into two sub-groups—one group has a *B. napus* sequence more closely related to the *BrNHX6.1* sequence, while the other group includes only *B. oleracea* and *B. napus* sequences, possibly indicating both an A and C genome version of *BrNHX6.1* (Figure 6B). From this analysis, the *B. napus* sequences that are clearly closely related to *BrNHX6.1* and *BrNHX6.2* will be hereafter designated as *BnNHX6.1* and *BnNHX6.2*, respectively.

### Expression analysis of *BnNHX6.1* and *BnNHX6.2*

**Figure 7:**
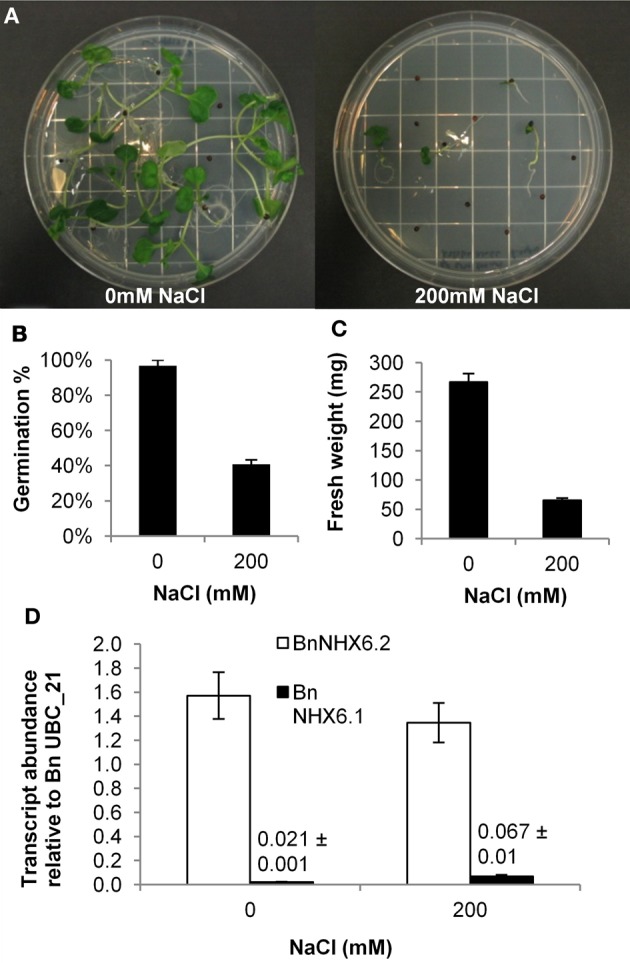
**Differential expression of BnNHX6.1 and BnNHX6.2 in *B. napus* seedlings in response to NaCl.** *B. napus* (cv Westar) seeds were sown on ½ strength MS media with or without 200 mM NaCl **(A)** and the germination percentage **(B)** and fresh weight per seedling **(C)** were compared between the two treatments. RNA was extracted from seedlings and the transcript abundance of BnNHX6.1 and BnNHX6.2 was assayed relative to the BnUBC_21 reference gene **(D)**. Data are the means of three biological replicates (±s.e.).

Having identified two *NHX6* genes in *B. napus*, we examined the relative expression of *BnNHX6.1* and *BnNHX6.2* in the presence and absence of salt stress. Firstly, to optimise the concentration of NaCl required to induce salt stress, we compared the germination percentage and fresh weight of *B. napus* cv. Westar seedlings grown in the presence and the absence of NaCl. The NaCl treatment had a marked effect on the germination and growth of *B. napus* seedlings (Figure 7A). Germination in the presence of NaCl was severely inhibited with only 41% of seeds germinating compared with 92% of seeds sown on plates lacking additional NaCl (Figure 7B). The mean fresh weight per seedling grown in the presence of NaCl was also greatly reduced compared to those grown in the absence of NaCl (Figure 7C). These results demonstrate that the NaCl treatment was sufficient to induce a severe stress impacting on the germination and growth of *B. napus* seedlings. We then examined the differential expression of both the *BnNHX6.1* and the *BnNHX6.2* genes in the presence and absence of the NaCl stress. The *BnNHX6.2* gene showed no significant difference in relative transcript abundance in response to the NaCl stress, whereas there was a significant increase in relative transcript abundance of the *BnNHX6.1* gene in response to the NaCl stress (Figure 7D). Interestingly, the relative expression of *BnNHX6.1* was extremely low compared to *BnNHX6.2* both in the presence and absence of NaCl (Figure 7D) suggesting that there may be some differential regulation of these two genes.

## Discussion

### Ortholog identification in *B. napus*

In this paper we have attempted to identify the *B. napus* orthologs of two *A. thaliana* proteins, AtNHX5 and AtNHX6. Although *A. thaliana* and *B. napus* are relatively closely related, precise ortholog identification is complicated by the recent WGT event in the *Brassica* species and the amphidiploid nature of the *B. napus* (AACC 2*n* = 38) genome. Consequently, there are potentially six *B. napus* homologs of *AtNHX5* and *AtNHX6* comprising three triplicated paralogs from both the A and C parental genomes.

Identification of *A. thaliana* homologs in the *Brassica* species has traditionally relied upon comparison to incomplete genomic sequences, generally in the form of bacterial artificial chromosome (BAC) clones. For example identification of *FLOWERING LOCUS C* (*FLC*) homologs in *B. rapa* entailed probing *B. rapa* BAC libraries to identify BAC clones containing co-linear regions (Yang et al., 1999). While successful in this case, this methodology was reliant on having all the appropriate co-linear region of the *B. rapa* genome present in the BAC library. The recent release of the *B. rapa* genome provides a complete *Brassica* genomic resource allowing *A. thaliana* gene orthologs to be identified with a high level of confidence.

Our search of the *B. rapa* genome identified only two *AtNHX6* orthologs. Analysis of the *B. rapa* genome has shown that there has been significant loss of gene paralogs in the three sub genomes since the WGT event (Wang et al., 2011), which is likely to be the reason that there is no *AtNHX6* ortholog on the MF1 sub genome. Interestingly, we could not find any evidence of an *AtNHX5* homolog in the *B. rapa* genome. While this came as a surprise, recent evidence shows that *AtNHX5* and *AtNHX6* act redundantly in *A. thaliana* (Bassil et al., 2011), and therefore the the *Brassica* species may not require a functional *AtNHX5* ortholog. Our analysis of the microsynteny around *AtNHX5* and its co-linearity with the three corresponding sub genomes in *B. rapa* indicated that a large block of *A. thaliana* genes including *AtNHX5* were missing from all three co-linear *B. rapa* sub genomes as a result of inter-chromosomal translocations in the LF and MF2 sub genomes and a fragment deletion in the MF1 sub-genome. The identification of an *AtNHX5* ortholog in the closely related *T. parvula* species indicates that there was an *AtNHX5* ortholog present in the ancient *Brassica* ancestral species prior to the Br—α WGT event. The loss of *AtNHX5* in the *Brassica* species could only have occurred either due to a localized deletion event occurring in the ancestral *Brassica* genome after the *T. parvula*-*Brassica* divergence, but before the *Brassica* WGT event or due to the loss of all three *AtNHX5* genes following the WGT event. It seems more likely that the *AtNHX5* orthologs were lost after the Br—α WGT event due to the differential disruption in the syntentic region around *AtNHX5* in the three *B. rapa* sub-genomes. If *AtNHX5* had been lost prior to the Br—α WGT event it would be reasonable to expect that the syntenic region in all three *B. rapa* sub-genomes would display a common chromosomal disruption that was then triplicated as part of the Br—α WGT event.

Sequencing of orthologous BAC clones of the *B. rapa* A genome, the *B. oleracea* C genome and the A and C genome components of *B. napus* have shown essentially complete co-linearity between the protein coding genes in the A and C genomes and sequence identity of 94–97% (Cheung et al., 2009). It could, therefore, be anticipated that the *B. oleracea* C genome is also likely to only have two *AtNHX6* orthologs. Our sequence analysis in *B. oleracea* and *B. napus* could only identify two distinct *AtNHX6* homologs that were also homologous to the *BrNHX6.1* and *BrNHX6.2* genes. It is important to note however, that all of the *B. oleracea* and *B. napus* sequences analysed in this phylogeny were derived from mRNA; it is therefore possible that additional *B. oleracea NHX6* homologs are present in the genome but have not been detected in the limited mRNA sequencing described here. The complete complement of *B. oleracea NHX6* homologs could only be resolved with a completed *B. oleracea* genome. The phylogenetic analysis of homologous *Brassica NHX6.1* sequences shows two distinct groupings, one including *B. rapa NHX6.1* and a *B. napus* sequence, and the other containing only *B. oleracea* and *B. napus* sequences. This provides evidence of possible A and C genome versions of *Brassica NHX6.1*. Unfortunately, we do not have a corresponding *B. oleracea NHX6.2* sequence available, making it difficult to clearly identify the A and C genome versions of *NHX6.2*. It is therefore most likely that there are two *AtNHX6* homologs in both *B. rapa* and *B. oleracea* and, as a consequence, four *AtNHX6* homologs in *B. napus*.

### Expression patterns of *BnNHX6.1* and *BnNHX6.2*

The analysis of the *B. rapa* genome showed that of the eight Arabidopsis *NHX* genes only *AtNHX1* and *AtNHX6* have retained multiple paralogs since the most recent triplication event. Gene duplications arising from polyploidy events are thought to be a major source of novel variation and gene function (Moore and Purugganan, 2003) and duplicated genes may be lost, develop new functions or share the functions of the original gene through differential expression or regulation (Blanc and Wolfe, 2004). It may be the case that the two orthologs of *AtNHX6* may have different functions or expression patterns. As the coding regions of all the identified *Brassica* NHX6 genes are highly homologous with *AtNHX6* and each other, it is likely that their functions (Na^+^/K^+^–H^+^ antiporting) remain identical. The analysis of the *BrNHX6.1* and *BrNHX6.2* promoter regions highlighted a significant region of similarity 300 bp upstream of the ATG. The conserved MYCCONSENSUS element is often associated with genes responsive to abiotic stress, such as the stress hormone abscisic acid (ABA), dehydration-responsive RD22, and cold responsive genes CBF, DREB1, and ICE1 (Chinnusamy et al., 2004). This is highly interesting given the potential role for *NHX* proteins in improving salt tolerance. Additionally, the equivalent EBOXNNAPA motif was found to be an essential promoter element for high expression of the napA storage protein in *B. napus* seeds (Stalberg et al., 1996). The conservation of these regulatory elements suggests conservation of functionality between AtNHX6 and its *B. rapa* homologs.

The expression of the *B. napus NHX6.2* gene is significantly higher than the *B. napus NHX6.1* gene in two week old seedlings, indicating that in this tissue type at least there does appear to be some differential expression. While there is no significant difference in expression of *BnNHX6.2* in response to NaCl, the expression of *BnNHX6.1* increased approximately three-fold in response to NaCl again highlighting differences in expression. It should be noted that the relative expression of *BnNHX6.1* was 20 times lower than the expression of *BnNHX6.2* in the NaCl treatment, and is suggestive of a more prominent role for *BnNHX6.2*. This is in contrast to the situation in *A. thaliana*, where the largest difference in expression observed is a 3.5 increase of AtNHX5 transcript in leaf tissue (Bassil et al., 2011). The two Brassica NHX6 isoforms do not appear to have different functions, but the expression of BnNHX6.1 is significantly lower than that of *BnNHX6.2*. This may indicate that the *BnNHX6.1* gene is simply sitting in the genome waiting to be lost, like most of the other triplicated paralogs of the *BrNHX* gene family. It would be of great interest to examine the expression patterns of *BnNHX6.1* and *BnNHX6.2* in a variety of tissues types in the presence and absence of NaCl to better elucidate differential expression patterns.

In this study we have demonstrated the use of the *B. rapa* genome in the successful identification of two *B. rapa* NHX6 orthologs, and used this information to identify two unique *B. napus* NHX6 orthologs. Our analysis also strongly suggests that there are four *B. napus* orthologs, comprising an A and C genome version of *BrNHX6.1* and *BrNHX6.2*. This study also indicates that there may be some differential expression of the *BnNHX6.1* and *BnNHX6.2*.

### Conflict of interest statement

The authors declare that the research was conducted in the absence of any commercial or financial relationships that could be construed as a potential conflict of interest.
